# Non-Destructive Sampling of Ancient Insect DNA

**DOI:** 10.1371/journal.pone.0005048

**Published:** 2009-04-01

**Authors:** Philip Francis Thomsen, Scott Elias, M. Thomas P. Gilbert, James Haile, Kasper Munch, Svetlana Kuzmina, Duane G. Froese, Andrei Sher, Richard N. Holdaway, Eske Willerslev

**Affiliations:** 1 Centre for Ancient Genetics and Environments, Natural History Museum and Institute of Biology, University of Copenhagen, Copenhagen, Denmark; 2 Geography Department, Royal Holloway, University of London, Egham, Surrey, United Kingdom; 3 Henry Wellcome Ancient Biomolecules Centre, Department of Zoology, University of Oxford, Oxford, United Kingdom; 4 Department of Integrative Biology, University of California, Berkeley, California, United States of America; 5 Department of Earth and Atmospheric Sciences, University of Alberta, Edmonton, Alberta, Canada; 6 Severtsov Institute of Ecology and Evolution, Russian Academy of Sciences, Moscow, Russia; 7 Palaecol Research Ltd, Christchurch, New Zealand; 8 School of Biological Sciences, University of Canterbury, Christchurch, New Zealand; American Museum of Natural History, United States of America

## Abstract

**Background:**

A major challenge for ancient DNA (aDNA) studies on insect remains is that sampling procedures involve at least partial destruction of the specimens. A recent extraction protocol reveals the possibility of obtaining DNA from past insect remains without causing visual morphological damage. We test the applicability of this protocol on historic museum beetle specimens dating back to AD 1820 and on ancient beetle chitin remains from permafrost (permanently frozen soil) dating back more than 47,000 years. Finally, we test the possibility of obtaining ancient insect DNA directly from non-frozen sediments deposited 3280-1800 years ago - an alternative approach that also does not involve destruction of valuable material.

**Methodology/Principal Findings:**

The success of the methodological approaches are tested by PCR and sequencing of COI and 16S mitochondrial DNA (mtDNA) fragments of 77–204 base pairs (-bp) in size using species-specific and general insect primers.

**Conclusion/Significance:**

The applied non-destructive DNA extraction method shows promising potential on insect museum specimens of historical age as far back as AD 1820, but less so on the ancient permafrost-preserved insect fossil remains tested, where DNA was obtained from samples up to ca. 26,000 years old. The non-frozen sediment DNA approach appears to have great potential for recording the former presence of insect taxa not normally preserved as macrofossils and opens new frontiers in research on ancient biodiversity.

## Introduction

Most ancient genetic studies have focused on vertebrates, plants and to a lesser extent microbes revealing aDNA research as a powerful tool for testing hypotheses in biology [1], [2]. Although insects are the most diverse animal group on Earth with more than 1 million described species, aDNA studies on insects have so far been limited and restricted largely to museum specimens of historical age, up to ca. 100 years [3], [4], [5], [6], or to geologically-ancient amber-entombed specimens millions of years old (e.g. [7], [8], [9]). While the former have produced exciting results relating to events in the near past, the latter have proved a classical example of how a lack of appropriate contamination controls in aDNA research may produce false positive results [10], [11].

Only three studies appear to have investigated insect DNA survival between these two extreme time-ranges: [12] studied grasshoppers from glacial deposits in Wyoming deposited ca. 400 years ago; [13] investigated beetle remains from ca. 20,000-year-old packrat middens from Texas; [14] studied 450,000- to 800,000-year-old silty-ice from the base of a Greenland ice core. All three studies gave positive results for the presence of insect DNA, which encourages further research on the possibilities of obtaining insect aDNA in other contexts.

Intriguingly, a major constraint on the use of historical, and particularly ancient, insect specimens in aDNA research is the destructive nature of the sampling procedure [15]. Obviously, this is a problem related to many aDNA sources, but is of particular concern with small specimens, such as insects, where even limited sampling may destroy important morphological characters. All the above insect ancient genetic studies have suffered from such destructive sampling procedures. One potential solution is the application of an extraction protocol that uses digestion buffers designed to enable the recovery of DNA from insect remains without causing visual external morphological damage to the material [16]. This method has been used successfully on museum specimens of beetles collected between 1952 and 2002.

Here, we report the results of a study that tested the potential of obtaining authentic ancient insect mtDNA using this non-destructive extraction procedure on historical museum beetle specimens dating back to AD 1820 and on ancient chitin from beetle macrofossils from permafrost dating back more than 47,000 years (DNA obtained from samples up to ca. 26,000 years old). Additionally, encouraged by the findings of insect aDNA in the Greenland silty-ice, we explored non-frozen sediments from New Zealand laid down between 3280 and 1800 years ago as a direct source of ancient insect DNA, even though no visible fossil insect remains were present. This non-frozen sediment DNA approach is interesting in the current context, as it holds the potential of obtaining insect aDNA without the destruction of valuable specimens, as well as providing data on former biodiversity in the absence of macrofossils and unobtainable in any other way.

## Results

The non-destructive DNA extraction procedure was tested on two types of samples: i) Twenty museum specimens (representing five different species) of beetles collected between AD 1820 and AD 2006 (the oldest historical museum insect remains from which DNA survival has been investigated), and ii) fourteen beetle macrofossils (chitin) from the late Pleistocene (ca. 47,600–20,100 ^14^C years BP) and late Pleistocene-early Holocene (ca. 10,595–7,110 ^14^C years BP). These macrofossils were recovered from permafrost sediments in Chukotka (Siberian Far East) and central Alaska in 2004 and 2005, respectively.

**Figure 1:**
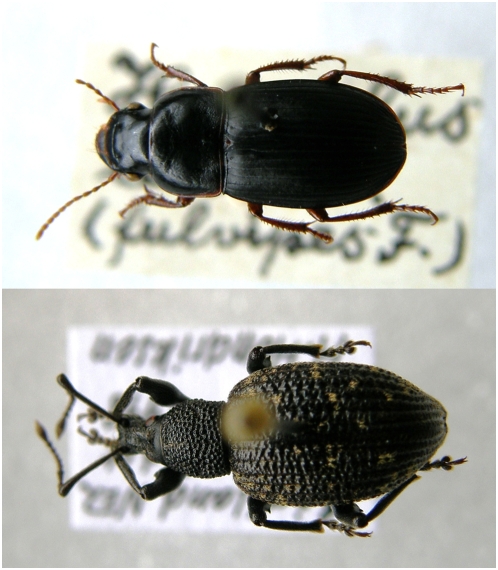
Museum samples post-extraction. Photographs of a) *Harpalus latus*, CFx7.2 and b) *Otiorhynchus sulcatus*, CFx7.16 after overnight treatment in the extraction buffer. No visual damage is seen on the specimens.

All twenty specimens of museum beetles produced amplifiable and authentic COI mtDNA sequences between 77–204 -bp in size. These were from the ground beetle *Harpalus latus* (Linnaeus, 1758), the pill beetle *Byrrhus pilula* (Linnaeus, 1758), the leaf beetle *Chrysolina polita* (Linnaeus, 1758) and two weevils, *Otiorhynchus sulcatus* (Fabricius, 1775) and *Curculio pyrrhoceras* Marsham, 1802 (Table 1). Of the 14 permafrost-preserved beetle chitin macrofossils, only three yielded successful COI or 16S mtDNA amplification products; a weevil *Lepidophorus thulius* (Kissinger, 1974) (ca. 10,595 ^14^C years BP, dated by association with wood from the sample), a ground beetle *Amara alpina* (Paykull, 1790) and a rove beetle *Tachinus brevipennis* Kiesenwetter, 1850, both with radiocarbon ages of ca. 26,000 ^14^C years BP, estimated from a sedimentation rate based on overlying and underlying radiocarbon dated samples of plant macrofossils (Table 2). The amplification products from the macrofossil remains were between 91–159-bp in size. An inverse relationship between amplification strength and length typical of aDNA supports the authenticity of the findings as does sequence identification in agreement with the morphological based taxonomic affiliation of the specimens (Tables 1 and 2). Importantly, none of the insect specimens subjected to DNA extraction seemed to have undergone any visible physical alterations after the extraction procedure (Figure 1).

**Table 1:** Historical museum specimens investigated in the study.

Sample #	Species	Family	Collected (A.D)	Size of amplified COI mtDNA		Locality	p.p.	Level
				78 bp.	204 bp.			
CFx7.1	*Harpalus latus* (Linnaeus, 1758)	Carabidae	1825	x	x	Jylland	98%	Species
CFx7.2			1899	x	x	Silkeborg	98%	Species
CFx7.3			1939	x	x	Grib skov	97%	Species
CFx7.4			2004	x	x	Ekkodalen, B.	96%	Genus
					143 bp.			
CFx7.5	*Byrrhus pilula* (Linnaeus, 1758)	Byrrhidae	1820		x	Jylland	100%	Species
CFx7.6			1930		x	Samsø	100%	Species
CFx7.7			1973		x	Tipperne	100%	Species
CFx7.8			2005		x	Brorfelde	100%	Species
				94 bp.	143 bp.			
CFx7.9	*Chrysolina polita* (Linnaeus, 1758)	Chrysomelidae	1899	x		Donse	95%	Family
CFx7.10			1942	x		Karlslunde strand	98%	Family
CFx7.11			1971	x	x	Snave, Fyn	95%	Family
CFx7.12			2006	x	x	Isenbjerg	95%	Family
				98 bp.	162 bp.			
CFx7.13	*Otiorhynchus sulcatus* (Fabricius, 1775)	Curculionidae	1884		x	Ålborg	95%	Genus
CFx7.14			1920	x	x	Christianssæde	94%	Species
CFx7.15			1970	x	x	Rørvig, Sjælland	94%	Species
CFx7.16			1998	x	x	Hillerød	94%	Species
				77 bp.	167 bp.			
CFx7.17	*Curculio pyrrhoceras* Marsham, 1802	Curculionidae	1896	x	x	Ålbek	86%	Species
CFx7.18			1911	x		Fønstrup	93%	Species
CFx7.19			1958	x	x	Hønning Pl.	95%	Species
CFx7.20			2000	x	x	Stenrand, Sj.	93%	Species

All specimens were collected in Denmark.

x) Authentic DNA sequence obtained; p.p.) posterior probability of assigning the sequence to the given taxonomic level.

**Table 2:** Ancient macrofossil remains investigated in the study.

Sample #	Species	Order	Family	Locality	Sediment	Age (^14^C yr. BP)	Collection year	p.p.	Level
CFx3.1	*Lepidophorus lineaticollis* Kirby, 1837	Coleoptera	Curculionidae	Alaska	Al-4-05-B24/QIII	47,600±1900 (UCIAMS 56391)	2005		
CFx3.2	Diptera indet ?!	Diptera	?	Alaska	Al-3-05-B9/QIV	9,752±85 (AA52066)	2005		
CFx3.3	*Lepyrus* sp. (a leg)	Coleoptera	Curculionidae	Alaska	Al-3-05-B9/QIV	9,752±85 (AA52066)	2005		
CFx3.4	*Lepidophorus thulius* (Kissinger, 1974)	Coleoptera	Curculionidae	Alaska	Al-2-05-B7/QIII	10,595±25 (UCIAMS 36670)	2005	100%	Order
CFx3.5	*Lepidophorus lineaticollis* Kirby, 1837	Coleoptera	Curculionidae	Alaska	Al-2-05-B7/QIII	10,595±25 (UCIAMS 36670)	2005		
CFx3.6	*Camponotus herculeanus* (Linnaeus, 1758)	Hymenoptera	Formicidae	Alaska	Al-3-05-B13/QIV	7,110±110 (GSC 6675)	2005		
CFx3.7	*Morychus* sp. nov?	Coleoptera	Byrrhidae	Alaska	Al-4-05-B24/QIII	47,600±1900 (UCIAMS 56391)	2005		
CFx3.8	*Amara alpina* (Paykull, 1790)	Coleoptera	Carabidae	Chukotka	ChM-B15	Ca. 26,000	2004	95%	Family
CFx3.9	*Lepyrus nordenskioeldi* Faust, 1885	Coleoptera	Curculionidae	Chukotka	ChM-B15	Ca. 26,000	2004		
CFx3.10	*Morychus viridis* Kuzmina & Korotyaev, 1987	Coleoptera	Byrrhidae	Chukotka	ChM-B15	Ca. 26,000	2004		
								63%	Species
CFx3.11	*Tachinus brevipennis* Kiesenwetter, 1850	Coleoptera	Staphylinidae	Chukotka	ChM-B15	Ca. 26,000	2004	73%	Family
CFx3.12	*Aphodius* sp nov?	Coleoptera	Scarabaeidae	Chukotka	ChM-B34	Ca. 20,900	2004		
CFx3.13	*Sitona borealis* Korotyaev, 1979	Coleoptera	Curculionidae	Chukotka	ChM-B33	Ca. 20,100	2004		
CFx3.14	*Notiophilus aquaticus* (Linnaeus, 1758)	Coleoptera	Carabidae	Chukotka	ChM-B15	Ca. 26,000	2004		

Authentic DNA sequence obtained from: CFx3.4, CFx3.8 and CFx3.11.

^14^C yr. BP) radiocarbon years before present (1950); p.p.) posterior probability of assigning the sequence to the given taxonomic level. The species *Amara alpina* is also known as *Curtonotus alpinus* (Paykull, 1790) in some literature. *Lepidophorus thulius* was until recently known as *Vitavitus thulius* Kissinger, 1974.

Alaska sample ages estimated from radiocarbon dates of associated plant macrofossil ages from sampling horizon. Chukotka sample ages estimated from sedimentation rate based on overlying and underlying radiocarbon dated samples of plant macrofossils (See Materials and Methods section).

In addition to the above, we examined the potential for long-term survival of insect DNA in temperate sediments. Two insect COI mtDNA sequences of 96-bp in length were obtained from one of the two non-frozen sediment samples from New Zealand. The sediments were laid down between 3280 and 1800 years BP [17]. The sequences were identified as being from a beetle and a moth/butterfly, respectively (Table 3).

**Table 3:** Non-frozen sediment samples investigated in the study.

Sample #	Locality	Age	p.p.	Level
ABC10652	Hukanui Pool, layer C, New Zealand	3280–1800 yr. BP	81%	Order: Lepidoptera
ABC10652	Hukanui Pool, layer C, New Zealand	3280–1800 yr. BP	85%	Order: Coleoptera
ABC10653	Hukanui Pool, layer F*, New Zealand	AD 1870-present		

Authentic DNA sequence obtained only from sample #ABC10652.

yr. BP) calendar years before present (1950); p.p.) posterior probability of assigning the sequence to the given taxonomic level.

* )This layer is mostly sheep faeces, see [21].

## Discussion

The 100% success rate on the beetle specimens from museum collections dating back 188 years suggests that the non-destructive extraction procedure tested has considerable potential for sampling historical insect material, even when more than 100 years older than the specimens originally tested with the method [16]. It may be worth exploring if similar success can be obtained on insect groups other than beetles, such as Lepidoptera, Diptera and Hymenoptera, whose chitinous exoskeleton is not as thick and resilient as that of beetles. However, we see no obvious reason why the procedure should not work on a variety of taxa. The result is significant in that museum insect specimens have already proved to be an important resource for e.g. identifying recent bottlenecks [4] or the development of traits such as insecticide resistance [5] etc. In particular, the non-destructive extraction procedure appears to have removed the need for destructive sampling.

The limited success of 3/14 (ca. 21%) on the truly ancient beetle chitin remains may result from either the remains no longer containing amplifiable endogenous DNA despite preservation in ideal frozen conditions for most of the preservation period (e.g. see [18]), or the extraction procedure not being efficient enough to retrieve DNA from truly ancient remains even where destructive sampling could have been successful. The possibility of a lower extraction efficiency is supported by the results of a similar non-destructive DNA extraction protocol for mammalian teeth [19]: only specimens that had been in museum collections for relatively short times yielded DNA using the non-destructive sampling method and remains that had been in collections for much longer periods gave products only with destructive sampling strategies. It appears that only limited success can be expected using the method of [16] on truly ancient insect specimens.

Interestingly, DNA from a beetle and a moth/butterfly was obtained from one of the two New Zealand temperate sediment samples, even in the absence of visible macrofossil material. The failure to obtain insect DNA from one of the two samples could result from spatial differences in the distribution of DNA source material. The success of the New Zealand non-frozen sediment DNA compared to the permafrost preserved macrofossils is surprising in that, although the sediment samples were several thousands of years younger than the macrofossils examined, it is generally believed that it is the temperature of preservation rather than the age itself that determines the level of DNA degradation [1]. The source of insect DNA preserved in the sediments may include material other than macrofossil remains of adults, such as eggs or larvae, additional to that of harder, chitinous materials. The results from the sediments are important because this is the first time insect DNA has been retrieved directly from non-frozen sediments. The approach may have wide applications. Ancient sediment-preserved DNA studies could reveal the former presence of taxa not normally preserved in the fossil record such as soft-bodied insects. Although the non-frozen sediment DNA approach involves destructive sampling, it has the advantage that the material is the sediment itself, which is usually abundant, and normally not too valuable to process.

## Materials and Methods

### Historical Museum Specimens

Four individuals each of five beetle species (a total of 20 specimens) were selected, to cover a historic period spanning from AD 1820 until today (Table 1). All specimens were collected in Denmark, and are held in the collection of the Natural History Museum, Copenhagen, Denmark. Sequences of the COI gene for all the five species were available on GenBank, which allowed the construction of species-specific primers (Table S1).

### Macrofossils

Fourteen macrofossils were recovered from permafrost sediments: 7 macrofossils from central Alaska and 7 from Main River, Ice Bluff (ledovy Obryv), Chukotka, northeastern Siberia (Table 2).

Co-ordinates for Alaska samples: 65°06′N, 153°17′W (sample# CFx3.1 and 3.7), 66°14′N, 148°57′W (sample# CFx3.4 and 3.5) and 65°59′N, 148°57′W (sample# CFx3.2, 3.3 and 3.6).

Co-ordinates for Chukotka samples: 64°06′N, 171°11′E.

All samples were kept in 96% ethanol in the freezer until non-destructive DNA extraction. Before extraction, samples were dried overnight at 55°C for the ethanol to evaporate. New primers for insects were constructed and additional primers from the literature were used [14], [20] to amplify COI and 16S mtDNA sequences, and the same sets of primers were used on all macrofossil samples (Table S1).

### Sediments

DNA from two samples of cave sediment from the late Quaternary Hukanui Pool site, eastern North Island, New Zealand [17], [21] was extracted and assayed as described in [22] and [23] (Table 3). The site was deposited beneath large erratic limestone blocks, and contained sediment layers between layers of well-dated volcanic tephras originating from the Taupo Volcanic Zone, 100 km to the west. The sediment was deposited between the Waimihia eruption of ca. 3280 years BP [24], and the AD 1870 surface, and the specific sediment sample from this study is ca. 3280-1800 years old. Primers from [14] were used to amplify COI mtDNA sequences (Table S1).

### DNA Extraction and PCR

DNA extraction and PCR setup was carried out in dedicated aDNA clean-laboratories [2]. DNA was extracted from museum specimens and macrofossils using the non-destructive method [16]: Whole specimens were placed in 2 ml Eppendorf Biopur tubes, fully immersed in digestion buffer (volume dependent on specimen size, 0.5–1.5 ml in this study), and incubated overnight at 55°C with gentle agitation. The buffer consisted of 3 mM CaCl_2_, 2% sodium dodecyl sulphate (SDS), 40 mM dithiotreitol (DTT), 250 mg/ml proteinase K, 100 mM Tris buffer pH 8 and 100 mM NaCl (final concentrations). After incubating with gentle agitation for 16–20 hours, specimens were removed from the buffer, placed in 100% EtOH for 2–4 hours to stop further digestion, air-dried, and replaced in their collections. Nucleic acids were purified from the digestion buffer using a Qiagen PCR purification kit (QIAquick).

DNA from the sediments was extracted using the procedure described in [22] and [23].

PCR reactions for all samples, except the sediments (see [22]), were the following: 1 µl DNA, 2.5 µl of each primer (10 µM), 2.5 µl 10× HiFi Buffer, 2 µl BSA, 1 µl MgSO_4_, 0.2 µl dNTPs and 0.1 µl Platinum Taq HiFidelity Polymerase enzyme (invitrogen, Carlsbad, CA) and 13.2 µl ddH_2_O giving a total 25 µl PCR reaction. PCR conditions were: 94°C for 2 min. followed by 60 cycles of 94°C for 30 sec., 50–52 °C for 30 sec., 68°C for 40 sec., completed with a final 68°C for 7 min. PCR products were tested on 2% Agarose gels stained with ethidium bromide. The amplified PCR products were purified using an Invitek purification kit (PCRapace) and cloned with Invitrogen Topo TA cloning kit. All PCR products were cloned prior to sequencing in order to ensure sequence accuracy. New PCRs were performed on 8–24 *E. coli* colonies, using the primers M13F and M13R and amplified for 35 cycles with annealing temperature of 54°C. PCR products containing the inserted PCR extracts were purified using vacuum suction and commercially sequenced (Macrogen, Seoul, Korea). If sequence differences were obtained from individuals of the same species, the sequence results were replicated to test for miscoding lesion damage [25], [26].

### Sequence Identification

All sequences were identified by a method using Bayesian approach to statistical assignment [27]. The method has advantages compared to the online BLAST search tool, by including phylogenetic information and providing statistically meaningful measures of confidence (posterior probabilities) to the taxonomic assignment.

### Radiocarbon dating

The age of the insect fossil remains were estimated from associated radiocarbon ages from *in situ* plant macrofossils from the same sampling horizons using Accelerator Mass Spectrometry (AMS) or radiometric (conventional) radiocarbon dating (Table 2). All ages are reported in radiocarbon years BP, which are slightly younger than their corresponding calendar year ages. Chukotka sample ages were estimated from sedimentation rate based on the following ages (^14^C years BP) of overlying and underlying radiocarbon dated samples of plant macrofossils: 33190±240 (OxA-15347), 29780±210 (OxA-14928), 28190±160 (OxA-15348), 25440±130 (OxA-14957), 22960±120 (OxA-15348), 21050±100 (OxA-14929), 20830±90 (OxA-15667) and 19850±80 (OxA-15668).

The New Zealand samples investigated are from a series taken from sediments between independently dated tephra (volcanic ash) beds laid down ca. 3280–1800 years BP as described in [17] and [21].

## Supporting Information

Table S1 Primer and amplification details. All PCRs: 50°C annealing temp. exept insCOIF/R: 52°C. All primers were HPLC purified.
(0.04 MB XLS)

## References

[1] Willerslev E, Hansen AJ, Poinar HN (2004). Isolation of nucleic acids and cultures from fossil ice and permafrost.. TRENDS Ecol Evol.

[2] Willerslev E, Cooper A (2005). Ancient DNA.. Proc R Soc B.

[3] Goldstein PZ, Desalle R (2003). Calibrating phylogenetic species formation in a threatened insect using DNA from historical specimens.. Mol Ecol.

[4] Harper GL, Maclean N, Goulson D (2006). Analysis of museum specimens suggests extreme genetic drift in the adonis blue butterfly (*Polyommatus bellargus*).. Biol J Linn Soc.

[5] Hartley CJ, Newcomb RD, Russell RJ, Yong GG, Stevens JR (2006). Amplification of DNA from preserved specimens shows blowflies were preadapted for the rapid evolution of insecticide resistance.. Proc Natl Acad Sci USA.

[6] Watts PC, Thomspon DJ, Allen KA, Kemp SJ (2006). How useful is DNA extracted from the legs of archived-insects for micro-satellite based population genetic analysis?. J Insect Cons.

[7] Cano RJ, Poinar H, Poinar GO (1992a). Isolation and partial characterisation of DNA from the bee *Proplebeia dominicana* (Apidae: Hymenoptera) in 25–40 million year old amber.. Med Sci Res.

[8] Cano RJ, Poinar HN, Roubik DW, Poinar GO (1992b). Enzymatic amplification and nucleotide sequencing of portions of the 18S rRNA gene of the bee *Proplebeia dominicana* (Apidae: Hymenoptera) isolated from 25–40 million year old Dominican amber.. Med Sci Res.

[9] DeSalle R, Gatesy J, Wheeler W, Grimaldi D (1992). DNA sequences from a fossil termite in Oligo-Miocene amber and their phylogenetic implications.. Science.

[10] Austin JJ, Ross AJ, Smith AB, Fortey RA, Thomas RH (1997). Problems of reproducibility - does geologically ancient DNA survive in amber-preserved insects?. Proc Biol Sci.

[11] Hebsgaard MB, Phillips MJ, Willerslev E (2005). Geologically ancient DNA: Fact or artefact?. Trends Microbiol.

[12] Chapco W, Litzenberger G (2004). A DNA investigation into the mysterious disappearance of the Rocky Mountain grasshopper, mega-pest of the 1800s.. Mol Phyl Evol.

[13] Reiss RA (2006). Ancient DNA from ice age insects: proceed with caution.. Quat Sci Rev.

[14] Willerslev E, Cappellini E, Boomsma W, Nielsen R, Hebsgaard MB (2007). Ancient Biomolecules from Deep Ice Cores Reveal a Forested Southern Greenland.. Science.

[15] Mandrioli M (2008). Insect collections and DNA analyses: how to manage collections?. Museum Management and Curatorship.

[16] Gilbert MTP, Moore W, Melchior L, Worobey M (2007). DNA Extraction from Dry Museum Beetles without Conferring External Morphological Damage.. PLoS ONE.

[17] Holdaway RN, Roberts RG, Beavan-Athfield NR, Olley JM, Worthy TH (2002). Optical dating of quartz sediments and accelerator mass spectrometry ^14^C dating of bone gelatin and moa eggshell: a comparison of age estimates for non-archaeological deposits in New Zealand.. J Royal Soc New Zealand.

[18] Hansen AJ, Mitchel DL, Rønn R, Wiuf C, Paniker L (2006). Crosslinks rather than strand breaks determine access to ancient DNA sequences from frozen sediments.. Genetics.

[19] Rohland N, Siedel H, Hofreiter M (2004). Nondestructive DNA extraction method for mitochondrial DNA analyses of museum specimens.. Biotechniques.

[20] Simon C, Frati F, Beckenbach A, Crespi B, Liu H, Flook P (1994). Evolution, weighting, and phylogenetic utility of mitochondrial gene sequence and a compilation of conserved polymerase chain reaction primers.. Ann Entomol Soc Am.

[21] Holdaway RN, Beavan NR (1999). Reliable ^14^C AMS dates on bird and Pacific rat *Rattus exulans* bone gelatin, from a CaCO_3_-rich deposit.. J Royal Soc New Zealand.

[22] Haile J, Holdaway R, Oliver K, Bunce M, Gilbert MTP (2007). Ancient DNA Chronology within Sediment Deposits: Are Paleobiological Reconstructions Possible and Is DNA Leaching a Factor?. Mol Biol Evol.

[23] Willerslev E, Hansen AJ, Brand TB, Binladen J, Gilbert MTP (2003). Diverse plant and animal DNA from Holocene and Pleistocene sedimentary records.. Science.

[24] Froggatt PC, Lowe JL (1990). A review of late Quaternary silicic and some other tephra formations from New Zealand: their stratigraphy, nomenclature, distribution, volume, and age.. NZ J Geol Geophys.

[25] Hansen AJ, Willerslev E, Wiuf C, Mourier T, Arctander P (2001). Statistical evidence for miscoding lesions in ancient DNA templates.. Mol Biol Evol.

[26] Binladen J, Wiuf C, Gilbert MTP, Bunce M, Larson G (2006). Assessing the fidelity of ancient DNA sequences amplified from nuclear genes.. Genetics.

[27] Munch K, Boomsma W, Huelsenbeck JP, Willerslev E, Nielsen R (2008). Statistical Assignment of DNA Sequences using Bayesian Phylogenetics.. System Biol.

